# Trajectories of sickness absence and disability pension days among 189,321 white-collar workers in the trade and retail industry; a 7-year longitudinal Swedish cohort study

**DOI:** 10.1186/s12889-022-14005-y

**Published:** 2022-08-21

**Authors:** Kristin Farrants, Kristina Alexanderson

**Affiliations:** Division of Insurance Medicine, Department of Clinical Neuroscience, Karolinska Institutet, 171 77 Stockholm, Sweden

**Keywords:** Sick leave, Group-based trajectory models, White-collar workers, Trade and retail industry, Job demands/control

## Abstract

**Objective:**

1) identify different trajectories of annual mean number of sickness absence (SA) and disability pension (DP) days among privately employed white-collar workers in the trade and retail industries and 2) investigate if sociodemographic and work-related characteristics were associated with trajectory membership.

**Methods:**

A longitudinal population-based cohort register study of all white-collar workers in the trade and retail industry in 2012 in Sweden (*N* = 189,321), with SA and DP data for 2010–2016. Group-based trajectory analysis was used to identify groups of individuals who followed similar trajectories of SA/DP days. Multinomial logistic regression was used to determine associations between sociodemographic and work-related factors and trajectory membership.

**Results:**

We identified four trajectories of SA/DP days. Most individuals (73%) belonged to the trajectory with 0 days during all seven years, followed by a trajectory of few days each year (24%). Very small minorities belonged to a trajectory with increasing SA/DP days (1%) or to constantly high SA/DP (2%). Men had a lower risk of belonging to any of the three trajectories with SA/DP than women (OR Low SA/DP 0.42, 95% CI 0.41–0.44; Increasing SA/DP 0.34, 0.30–0.38; High SA/DP 0.33, 0.29–0.37). Individuals in occupations with low job control had a higher risk of belonging to the trajectory High SA/DP (OR low demands/low control 1.51; 95% CI 1.25–1.83; medium demands/low control 1.47, 1.21–1.78; high demands/low control 1.35, 1.13–1.61).

**Conclusion:**

Most white-collar belonged to trajectories with no or low SA/DP. Level of job control was more strongly associated with trajectory memberships than level of job demands.

**Supplementary Information:**

The online version contains supplementary material available at 10.1186/s12889-022-14005-y.

## Introduction

Sickness absence (SA) and disability pension (DP) have consequences for society, for insurance agencies, for employers, and for the individual [1, 2]. There is very little scientific knowledge about SA and DP among white-collar workers in the trade and retail industry, even though the trade and retail industry employs about 10% of those of working ages in activity in Sweden [3].

White-collar workers generally have lower SA rates than blue-collar workers [4, 5]. While most previous research has focused on groups with high SA rates, it is also important to gain knowledge about groups with lower rates, as they constitute large parts of the labour market, meaning that their SA has great implications for their companies, society, and themselves.

In many countries, women have higher SA rates than men [6]. Possible explanations for this are higher morbidity rates among women, especially the types of morbidity leading to SA [7, 8], that the scientific knowledge regarding diagnosing, treatment, prevention, and rehabilitation measures for women is more limited, or that women have more ergonomically or psychosocially demanding paid and unpaid work [9].

There have been some indications that sickness absence rates among white-collar workers increased in the years 2008 to 2020 [10], and that, at least in 2008 to 2015, this increase also occurred in the trade and retail industry [11]. However, these studies have used time series data, i.e., a new study base each measurement point, and this had not been studied using longitudinal cohort studies before. Studying group averages can also conceal within-group differences or sub-groups in the population that actually are distinct from each other [12, 13].

Previous research has shown associations between working environment and sickness absence [14–17]. In this project we use the job demands/control theory, first stated by Karasek and Theorell [16–18], where several studies have found an association between high demands/low control on the one hand, and cardiovascular diseases [19], sleeping disorders [20], or mental disorders such as depression or burnout [21–24]. There are also studies that have found associations between levels of demands and control and SA/DP [16–18]. However, there is very little knowledge about how psychosocial working environment is associated with patterns of SA/DP over time.

The trade and retail industry is a branch of industry with high staff turnover [25]. There is little knowledge about differences in SA/DP patterns among those who change occupation, branch of industry, or sector, and those who do not.

The aims of this study were to 1) identify different trajectories of annual mean number of SA and DP days among privately employed white-collar workers in the trade and retail industries and 2) to investigate if sociodemographic and work-related characteristics were associated with trajectory membership.

## Materials and methods

This is a population-based longitudinal cohort study of SA and DP among privately employed white-collar workers in the trade and retail industry during the years 2010–2016.

### Data and study population

We used data from three nation-wide Swedish administrative registers linked at individual level by use of the Personal Identity Number [26] (PIN, a unique ten-digit number assigned to all Swedish residents): (1) Longitudinal Integration Database for Health Insurance and Labour Market Studies (LISA by Swedish acronym) held by Statistics Sweden: to identify the source population and for information on age, sex, country of birth, type of living area, family situation, educational level, income, size of workplace, occupational code according to the Swedish Standard for Occupational Classification (SSYK by Swedish acronym, the Swedish version of the International Classification of Occupations, ISCO), sector, and branch of industry according to the Swedish Standard for Industrial Classification (SNI by Swedish acronym) in 2012–2016; (2) MicroData for Analysis of the Social Insurance database (MiDAS) held by the Social Insurance Agency: for information on SA spells > 14 days and DP (dates, extent (25, 50, 75, or 100% of full-time) for 2010–2016); (3) the Cause of Death Register held by the National Board of Health and Welfare: for information on date of death.

The study population was all those who were aged 18–67 years and registered as living in Sweden on both 31 December 2011 and 31 December 2012, had an occupational code according to SSYK that indicated a white-collar occupation, were employed at a private sector company in the trade and retail industry according to SNI, and during 2012 had income from work, parental benefits, and/or SA/DP that amounted to at least 7920 SEK (that is, 75% of the necessary income level to qualify for SA benefits from the Social Insurance Agency). The limit of 75% of the minimum income to qualify for SA benefits was set since in many cases SA benefits is about 75% of the work income; without this adjustment, people with low incomes and long-term SA might have fallen below the minimum income level to be included in the study [27]. Those who were employed in the public sector, self-employed, or who had full-time DP the whole year 2012 were excluded, and those who died or emigrated before 1 January 2017 were excluded. The final study population was 189,321 individuals.

### Public sickness absence insurance in Sweden

All people living in Sweden aged ≥ 16 years with an income from work or unemployment benefits, who due to disease or injury have a reduced work capacity, are covered by the national public SA insurance providing SA benefits. After a first qualifying day, the employer pays sick pay for the first 14 days of a SA spell, thereafter, SA benefits are paid by the Social Insurance Agency. Self-employed have more qualifying days. Unemployed get SA benefits from the Social Insurance Agency after the first qualifying day. A physician certificate is required after 7 days of self-certification. In this study, data on SA with benefits from the Social Insurance Agency were used. SA spells < 15 days were not included in the study, so as not to introduce bias regarding those who might have been unemployed part of the year. All residents in Sweden aged 19–64 years, whose work capacity is permanently or long-term reduced due to disease or injury, can be granted DP from the Social Insurance Agency.

Both SA and DP can be granted for part- or full-time (25, 50, 75, or 100% of ordinary work hours). SA benefits cover 80% and DP benefits 64% of lost income, both up to a certain level [28, 29]. We used net days of SA/DP so that partial days of SA/DP were combined, e.g., two days of part-time absence for 50% were summed to one net day. The first 14 days in SA spells were counted as being of the same extent as day 15 for the purpose of calculating net days. In all analyses SA and DP days were combined. Mean number of SA/DP net days/calendar year were calculated for each individual [28].

### Variables

We used information on the following variables from 2012 unless otherwise noted: *Sex*: woman or man; *Age:* 18–24, 25–34, 35–44, 45–54, 55–64, or 65–67 years; *Country of birth:* Sweden, other Nordic country, other EU25, or rest of world including missing; *Educational level:* compulsory school (≤ 9 years or missing), high school (10–12 years), or college/university (≥ 13 years); *Family situation:* married/cohabiting with children < 18 at home, married/cohabiting without children at home, single with children at home, or single without children at home; *Type of living area:* large city (Stockholm, Gothenburg or Malmö), medium-sized town > 90,000 inhabitants within 30 km of city centre), or small town/rural (< 90,000 inhabitants within 30 km of city centre or rural); *Job demands/control*: based on a job exposure matrix derived from the Swedish Work Environment Survey from Statistics Sweden [30], categorised based on tertiles into the following nine groups: high demands/high control, high demands/medium control, high demands/low control, medium demands/high control, medium demands/medium control, medium demands/low control, low demands/high control, low demands/medium control, low demands/low control (for more information on the job exposure matrix and the categorisation, see Norberg et al. [31]); *Having changed branch of industry in 2016,* based on SNI, categorised into the following six groups: trade and retail (no change), manufacturing, services, transport, construction and installation, care and education, or hospitality; *Having changed sector in 2016*: state, region, municipal, private company (no change), or other; *Change of occupation (based on SSYK code) 2012–2016:* no change or change within sub-major group (according to SSYK classifications), change of sub-major group within major group, change to higher major group (e.g., from major group 2 Professionals to major group 1 Managers), and change to lower major group (e.g., from major group 1 Managers to major group 2 Professionals).

### Statistical analyses

We used a procedure known as Group-Based Trajectory Modelling (Proc traj in SAS) to identify possible trajectories of mean SA/DP days/year in the seven years 2010–2016. Those who had 0 days during all of the seven studied years were categorized into a group of their own, since their trajectory was directly observable. We then fit a group-based trajectory model using only those who had any SA and/or DP during at least one of the seven years.

We determined the best-fitted model related to the number of trajectories using the Bayesian Information Criterion (BIC) [32], where values closer to 0 indicate better fit. The BIC value of a n trajectory model was compared to a n-1 trajectory model, and if the BIC value was smaller, the n group model was considered a better fit than the n-1 trajectory model. In order to keep the number of trajectories within reach for interpretation, a requirement of a minimum of 5% of the study population for the smallest trajectory was introduced (of the population used to build the model, i.e., excluding those who had 0 days each year, due to the size of this group relative to all others) [33]. We also used the method described by Coté et al. [34] to evaluate model fit, whereby the average probability for belonging to the trajectory should be above 0.70 in all trajectories. The final model was the one that fulfilled all the criteria of decreasing BIC value, at least 5% of the study population in each trajectory, and a ≥ 0.70 average probability of belonging to the trajectory in all trajectories.

In the next type of analyses, once the optimal number of trajectories was computed, the individual probabilities of belonging to a particular trajectory were estimated using a multinomial logit function. Individuals were assigned to the trajectory that they had the greatest probability of belonging to.

We used multinomial logistic regression to determine the association between each of the variables and belonging to each trajectory with crude and mutually adjusted odds ratios (OR) and 95% confidence intervals (CI). The reference groups for each respective variable were chosen as the largest groups, or the groups expected to have the lowest risk of belonging to trajectories other than “No SA/DP” (since ORs > 1 are easier to interpret).

Since those who are ≥ 65 years are not eligible for DP benefits (but can still be eligible for SA benefits), we ran sensitivity analyses where we excluded all those who were ≥ 61 years in 2012, and thus were ≥ 65 years during at least one of the follow-up years.

All methods were performed in accordance with the relevant guidelines and regulations. The project was approved of by the Regional Ethical Review Board of Stockholm. All analyses were done in SAS v 9.4.

## Results

Table 1 shows the sociodemographic characteristics of the cohort in 2012. There was a slightly higher proportion of men (55.56%) and more than half were aged 35–54.

**Table 1:** Sociodemographic and work**-**related information on the study cohort of privately employed white**-**collar workers in 2012

		**Total**		**Women**		**Men**	
		**n**	**%**	**n**	**%**	**n**	**%**
	**All **	**189,321**	**100**	**84,273**	**100**	**105,048**	**100**
*Sex*	Women	84,273	44.51				
	Men	105,048	55.49				
*Age*	18–24 years	8027	4.24	4421	5.25	3606	3.43
	25–34 years	40,281	21.28	19,977	23.71	20,304	19.33
	35–44 years	60,105	31.75	26,610	31.58	33,495	31.89
	45–54 years	49,564	26.18	20,571	24.41	28,993	27.6
	55–64 years	28,889	15.26	11,736	13.93	17,153	16.33
	65–67 years	2455	1.3	958	1.14	1497	1.43
*Type of living area*	Large city	97,733	51.62	45,530	54.03	52,203	49.69
	Medium-sized town	60,185	31.79	25,169	29.87	35,016	33.33
	Small town or rural	31,403	16.59	13,574	16.11	17,829	16.97
*Educational level (years)*	Elementary (0–9 years)	14,291	7.55	4683	5.56	9608	9.15
	High school (10–12 years)	98,496	52.03	41,860	49.67	56,636	53.91
	University/college (> 12 years)	76,534	40.43	37,730	44.77	38,804	36.94
*Country of birth*	Sweden	173,386	91.58	75,903	90.07	97,483	92.8
	Other Nordic country	4048	2.14	2220	2.63	1828	1.74
	Other EU-25	3290	1.74	1627	1.93	1663	1.58
	Rest of the world	8597	4.54	4523	5.37	4074	3.88
*Family situation*	Married/cohabiting without children	25,068	13.24	11,149	13.23	13,919	13.25
	Married/cohabiting with children	95,105	50.23	40,188	47.69	54,917	52.28
	Single without children	57,277	30.25	24,986	29.65	32,291	30.74
	Single with children	11,871	6.27	7950	9.43	3921	3.73
*Number of employees at workplace*	1–9 employees	51,168	27.03	23,981	28.46	27,187	25.88
	10–49 employees	74,965	39.6	30,696	36.42	44,269	42.14
	50–99 employees	22,371	11.82	9518	11.29	12,853	12.24
	100–499 employees	32,181	17.00	15,690	18.62	16,491	15.7
	≥ 500 employees	8636	4.56	4388	5.21	4248	4.04
*Job control/ demands*	Low control, low demands	21,650	11.44	16,617	19.72	5033	4.79
	Low control, medium demands	16,740	8.84	11,270	13.37	5470	5.21
	Low control, high demands	24,872	13.14	21,746	25.8	3126	2.98
	Medium control, Low demands	20,117	10.63	8755	10.39	11,362	10.82
	Medium control, medium demands	21,659	11.44	6478	7.69	15,181	14.45
	Medium control, high demands	21,374	11.29	12,323	14.62	9051	8.62
	High control, low demands	21,158	11.18	2361	2.8	18,797	17.89
	High control, medium demands	24,791	13.09	1624	1.93	23,167	22.05
	High control, high demands	16,960	8.96	3099	3.68	13,861	13.19

The majority among both women and men lived in large cities (Stockholm, Gothenburg, or Malmö), and were born in Sweden. A very small proportion (5.60% of women and 9.21% of men) had only elementary education, while 44.89% of women and 37.10% of men had at least some college/university education. The majority were married/cohabiting and having children < 18 years living at home, but more than twice the proportion of women (9.40%) than men (3.72%) were single parents with children living at home.

**Fig. 1:**
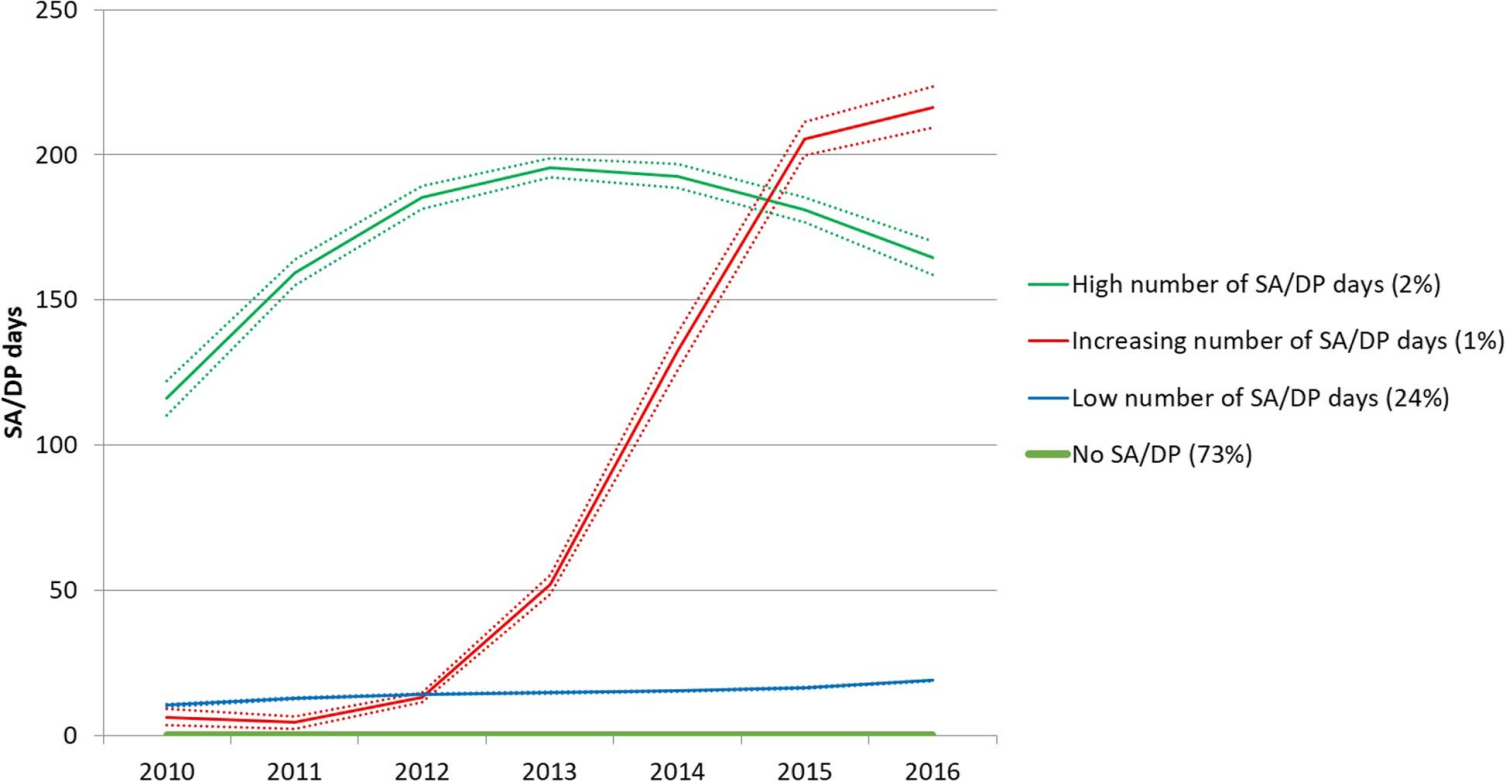
Four trajectories of sickness absence (SA)/disability pension (DP) from 2010–2016 among 189,321 white-collar workers in the trade and retail industry in 2012, and the proportion of the cohort that belong to each trajectory

We identified four trajectories in the model that fulfilled our criteria of decreasing BIC value as opposed to a more parsimonious model, at least 5% of the population used to build the model in each trajectory, and an average posterior probability of > 0.7 in each trajectory. The model with 5 trajectories led to two trajectories having less than 5% of the population each, although the BIC value continued to decrease. Figure 1 shows the four trajectories identified in the best-fitting model. The most common trajectory was that with No SA/DP throughout the seven studied years, which represented 73% of the study population, followed by the trajectory Low number of SA/DP days (24% of the study population). The trajectory High number of SA/DP days represented 2% of the study population, and the trajectory Increasing number of SA/DP days represented 1%.

Table 2 shows the distribution of individuals by sociodemographic and work-related characteristics. A higher proportion of women than men belonged to trajectories with SA/DP (Low SA/DP 32.25% women, 17.20% men; Increasing SA/DP 1.87% women 0.84% men; High SA/DP 2.49% women 0.86% men), whereas a higher proportion of men than women belonged to the trajectory no SA/DP (63.39% women, 81.09% men). A higher proportion of those with college/university education belonged to the trajectory No SA/DP (76.26%, compared to 71.53% among those with only high school education and 68.48% among those with only elementary education). A lower proportion of those who changed to the Care and education branch of industry belonged to the No SA/DP trajectory (57.31%) than any other branch of industry (range 65.31–76.25%).

**Table 2:** Distribution of sociodemographic and job**-**related characteristics in each of the 4 trajectories of white**-**collar workers in the trade and retail industry 2012 with different patterns of sickness absence (SA)/disability pension (DP) days/year over 2012–2016 identified by group**-**based trajectory modelling

	No SA/DP (*n* = 141 134)	Low SA/DP (*n* = 45 458)	Increasing SA/DP (*n* = 2469)	High SA/DP (*n* = 3016)
	**%**	**%**	**%**	**%**
*Total*	73.21	23.90	1.30	1.59
*Sex*				
Women	63.39	32.25	1.87	2.49
Men	81.09	17.20	0.84	0.86
*Age*				
18–24 years	77.82	21.13	0.74	0.31
25–34 years	73.13	25.23	1.06	0.58
35–44 years	74.82	22.65	1.29	1.24
45–54 years	72.75	23.64	1.49	2.12
55–64 years	68.72	26.42	1.58	3.28
65–67 years	82.40	17.52	0.04	0.04
*Type of living area*				
Large city	74.16	23.52	1.18	1.15
Medium-sized town	73.14	23.77	1.32	1.78
Small town or rural	70.41	25.36	1.63	2.60
*Education (years)*				
Elementary (0–9 years)	68.48	26.81	1.79	2.92
High school (10–12 years)	71.53	25.21	1.42	1.83
College/university (> 12 years)	76.26	21.67	1.04	1.03
*Birth country*				
Sweden	73.52	23.66	1.26	1.57
Other Nordic country	69.84	26.14	1.75	2.27
Other EU25	73.34	23.43	1.73	1.49
Rest of the world	68.64	27.96	1.70	1.70
*Family situation*				
Married/partner without children	70.73	25.18	1.36	2.73
Married/partner with children	74.79	22.88	1.16	1.17
Single without children	73.9	23.26	1.29	1.55
Single with children	62.49	32.47	2.34	2.70
*Demands/control*				
High demands/high control	78.84	19.41	0.94	0.80
High demands/medium control	71.01	25.89	1.49	1.61
High demands/low control	73.72	23.83	1.22	1.23
Medium demands/high control	80.46	17.89	0.87	0.78
Medium demands/medium control	75.41	22.08	1.13	1.37
Medium demands/low control	64.61	30.77	1.93	2.70
Low demands/high control	79.90	18.34	0.89	0.87
Low demands/medium control	73.72	23.83	1.22	1.23
Low demands/low control	67.53	28.60	1.55	2.32
*Changed branch of industry in 2016*				
Construction (*n* = 2337)	70.73	26.66	0.98	1.63
Hospitality (*n* = 896)	65.85	29.58	2.01	2.57
Manufacturing (*n* = 9274)	76.28	22.01	0.79	0.93
Unknown (*n* = 10,676)	65.31	23.03	4.76	6.89
Services (*n* = 24,103)	73.02	24.70	1.11	1.17
Transport (*n* = 1217)	69.76	27.61	0.90	1.73
Care and education (*n* = 6252)	57.31	38.10	1.92	2.67
Trade and retail (*n* = 134,566)	74.52	23.18	1.07	1.23
*Changed sector in 2016*				
Municipal (*n* = 3880)	58.02	37.50	2.27	2.22
Region (*n* = 1146)	59.25	37.70	1.48	1.57
State (*n* = 5717)	63.11	33.69	1.50	1.70
Other (*n* = 3477)	69.51	26.17	1.84	2.47
Private sector (*n* = 158,476)	74.96	22.95	1.00	1.09
*Change of occupation*				
Change within occupational category or no change (*n* = 93,999)	73.65	22.93	1.43	1.98
Change of occupational category within the same major occupational group (*n* = 43,372)	73.71	24.10	1.04	1.15
Change to a higher major occupational group (e.g. from 2 to 1) (*n* = 19,568)	76.82	21.55	0.87	0.76
Change to a lower major occupational group (e.g. from 1 to 2) (*n* = 32,382)	69.10	27.87	1.50	1.53
*Size of workplace*				
1–9 employees	71.56	24.28	1.60	2.56
10–49 employees	73.58	23.63	1.32	1.47
50–99 employees	73.88	24.18	0.97	0.97
100–499 employees	74.25	23.69	1.08	0.98
500 + employees	74.25	24.05	0.98	0.72

Table 3 shows crude and mutually adjusted odds ratios over associations between the covariates and belonging to each trajectory in a multinomial logistic regression.

**Table 3:** Crude and mutually adjusted odds ratios (OR) and 95% confidence intervals (CI) for the association between sociodemographic and work**-**related factors and belonging to respective trajectory group of sickness absence (SA)/disability pension (DP) days per year, compared to the trajectory group called No SA/DP

	Low SA/DP (*n* = 45 458)		Increasing SA/DP (*n* = 2469)		High SA/DP (*n* = 3016)	
	Crude OR (95% CI)	Adjusted OR (95% CI)	Crude OR (95% CI)	Adjusted OR (95% CI)	Crude OR (95% CI)	Adjusted OR (95% CI)
*Sex*						
Women	Ref	Ref	Ref	Ref	Ref	Ref
Men	0.42 (0.41–0.43)	0.42 (0.41–0.44)	0.35 (0.32–0.38)	0.34 (0.30–0.38)	0.27 (0.25–0.29)	0.33 (0.29–0.37)
*Age*						
18–24 years	0.90 (0.85–0.95)	0.75 (0.70–0.81)	0.55 (0.42–0.72)	0.35 (0.24–0.49)	0.24 (0.16–0.36)	0.09 (0.05–0.16)
25–34 years	1.14 (1.11–1.17)	1.11 (1.07–1.15)	0.84 (0.75–0.95)	0.81 (0.70–0.93)	0.48 (0.41–0.55)	0.40 (0.33–0.48)
35–44 years	Ref	Ref	Ref	Ref	Ref	Ref
45–54 years	1.07 (1.04–1.10)	1.09 (1.06–1.13)	1.19 (1.08–1.32)	1.13 (1.00–1.27)	1.76 (1.6–1.93)	1.87 (1.66–2.09)
55–64 years	1.27 (1.23–1.31)	1.34 (1.29–1.40)	1.34 (1.19–1.50)	1.36 (1.15–1.60)	2.87 (2.61–3.17)	2.40 (2.08–2.77)
65–67 years	0.70 (0.63–0.78)	0.65 (0.54–0.78)	0.03 (0.00–0.20)	0,03 (0,00- > 999,99)	0.03 (0.00–0.21)	0.06 (0.01–0.43)
*Type of living area*						
Large city	Ref	Ref	Ref	Ref	Ref	Ref
Medium-sized town	1.03 (1.00–1.05)	1.02 (1.00–1.05)	1.13 (1.03–1.24)	1.11 (0.99–1.24)	1.57 (1.44–1.71)	1.42 (1.27–1.58)
Small town or rural	1.14 (1.1–1.17)	1.1 (1.07–1.14)	1.45 (1.31–1.62)	1.44 (1.27–1.63)	2.39 (2.18–2.62)	1.85 (1.65–2.08)
*Education (years)*						
Elementary (0–9 years)	1.38 (1.32–1.44)	1.59 (1.52–1.67)	1.91 (1.66–2.2)	2.07 (1.73–2.46)	3.16 (2.8–3.57)	2.64 (2.25–3.1)
High school (10–12 years)	1.24 (1.21–1.27)	1.33 (1.29–1.36)	1.46 (1.33–1.59)	1.38 (1.25–1.54)	1.90 (1.74–2.06)	1.68 (1.51–1.86)
College/university (> 12 years)	Ref	Ref	Ref	Ref	Ref	Ref
*Birth country*						
Sweden	Ref	Ref	Ref	Ref	Ref	Ref
Other Nordic country	1.16 (1.08–1.25)	1.06 (0.98–1.14)	1.47 (1.16–1.86)	1.14 (0.85–1.54)	1.53 (1.24–1.89)	1.11 (0.85–1.45)
Other EU25	0.99 (0.92–1.08)	0.99 (0.91–1.08)	1.38 (1.06–1.80)	1.35 (0.98–1.86)	0.95 (0.72–1.27)	0.87 (0.60–1.27)
Rest of the world	1.27 (1.21–1.33)	1.27 (1.21–1.34)	1.45 (1.22–1.71)	1.59 (1.31–1.94)	1.16 (0.98–1.37)	1.49 (1.2–1.84)
*Family situation*						
Married/partner without children	Ref	Ref	Ref	Ref	Ref	Ref
Married/partner with children	0.86 (0.83–0.89)	0.97 (0.93–1.01)	0.8 (0.71–0.91)	0.91 (0.78–1.08)	0.40 (0.37–0.45)	0.79 (0.69–0.91)
Single, without children	0.88 (0.85–0.92)	0.98 (0.94–1.03)	0.9 (0.79–1.03)	1.07 (0.90–1.27)	0.54 (0.49–0.60)	1.14 (0.99–1.32)
Single with children	1.46 (1.39–1.53)	1.28 (1.21–1.35)	1.94 (1.66–2.28)	1.45 (1.18–1.77)	1.12 (0.98–1.28)	1.19 (0.99–1.42)
*Demands/control*						
High demands/high control	0.84 (0.80–0.88)	0.90 (0.85–0.94)	0.8 (0.65–0.98)	0.89 (0.71–1.13)	0.56 (0.46–0.69)	0.51 (0.39–0.66)
High demands/medium control	1.25 (1.19–1.30)	0.98 (0.94–1.03)	1.40 (1.18–1.65)	1.17 (0.96–1.42)	1.25 (1.07–1.46)	1.00 (0.82–1.21)
High demands/low control	1.63 (1.56–1.70)	1.01 (0.97–1.06)	1.99 (1.70–2.32)	1.19 (0.98–1.44)	2.30 (2.00–2.64)	1.35 (1.13–1.62)
Medium demands/high control	0.76 (0.73–0.80)	0.92 (0.87–0.96)	0.72 (0.60–0.86)	0.97 (0.77–1.21)	0.53 (0.44–0.64)	0.61 (0.48–0.77)
Medium demands/medium control	Ref	Ref	Ref	Ref	Ref	Ref
Medium demands/low control	1.41 (1.35–1.48)	1.03 (0.98–1.08)	1.60 (1.34–1.90)	1.11 (0.90–1.37)	2.07 (1.79–2.41)	1.47 (1.21–1.78)
Low demands/high control	0.78 (0.75–0.82)	0.92 (0.87–0.97)	0.74 (0.61–0.90)	0.96 (0.76–1.21)	0.60 (0.50–0.72)	0.66 (0.52–0.84)
Low demands/medium control	1.10 (1.05–1.16)	0.99 (0.94–1.04)	1.11 (0.93–1.32)	1.05 (0.85–1.3)	0.92 (0.77–1.09)	0.85 (0.69–1.06)
Low demands/low control	1.45 (1.38–1.51)	1.02 (0.97–1.07)	1.53 (1.29–1.80)	1.06 (0.86–1.3)	1.89 (1.64–2.19)	1.51 (1.25–1.83)
*Changed branch of industry in 2016*						
Construction	1.21 (1.10–1.33)	1.36 (1.23–1.50)	0.97 (0.64–1.47)	1.07 (0.69–1.68)	1.40 (1.01–1.93)	1.72 (1.21–2.44)
Hotell, restaurant	1.44 (1.25–1.67)	1.25 (1.06–1.46)	2.13 (1.33–3.41)	1.89 (1.14–3.12)	2.37 (1.56–3.60)	2.49 (1.59–3.91)
Manufacturing	0.93 (0.88–0.98)	1.05 (0.99–1.10)	0.72 (0.57–0.91)	0.84 (0.66–1.07)	0.74 (0.59–0.92)	0.83 (0.65–1.07)
Unknown	1.13 (1.08–1.19)	1.10 (0.93–1.30)	5.08 (4.58–5.64)	1.98 (1.24–3.18)	6.40 (5.85–7.01)	3.50 (2.44–5.02)
Sevices	1.09 (1.05–1.12)	1.10 (1.06–1.14)	1.06 (0.93–1.21)	1.12 (0.97–1.30)	0.97 (0.86–1.10)	1.22 (1.06–1.42)
Transport	1.27 (1.12–1.45)	1.37 (1.2–1.57)	0.90 (0.50–1.64)	1.12 (0.61–2.04)	1.50 (0.97–2.32)	1.70 (1.04–2.77)
Care and education	2.14 (2.03–2.26)	1.53 (1.42–1.65)	2.34 (1.93–2.82)	1.58 (1.22–2.06)	2.83 (2.40–3.33)	2.54 (2.02–3.19)
Trade and retail	Ref	Ref	Ref	Ref	Ref	Ref
*Changed sector in 2016*						
Municipal	2.11 (1.97–2.26)	1.32 (1.21–1.44)	2.94 (2.36–3.66)	1.65 (1.24–2.19)	2.63 (2.11–3.28)	0.98 (0.74–1.29)
Region	2.08 (1.84–2.35)	1.26 (1.10–1.45)	1.88 (1.16–3.05)	1.14 (0.67–1.94)	1.83 (1.14–2.92)	0.79 (0.47–1.32)
State	1.74 (1.65–1.85)	1.40 (1.32–1.48)	1.79 (1.44–2.23)	1.31 (1.04–1.64)	1.85 (1.51–2.28)	1.26 (1.02–1.56)
Other	1.23 (1.14–1.33)	1.07 (0.99–1.16)	1.99 (1.55–2.56)	1.62 (1.25–2.10)	2.45 (1.97–3.06)	1.61 (1.28–2.03)
Private enterprise	Ref	Ref	Ref	Ref	Ref	Ref
*Change of occupation*						
No change or change within sub-major group group	Ref	Ref	Ref	Ref	Ref	Ref
Change of sub-major group within major group	1.05 (1.02–1.08)	1.00 (0.97–1.03)	0.73 (0.65–0.81)	0.82 (0.73–0.93)	0.58 (0.52–0.64)	0.79 (0.71–0.89)
Change to higher major group	0.90 (0.87–0.94)	0.87 (0.83–0.9)	0.58 (0.50–0.69)	0.67 (0.56–0.80)	0.37 (0.31–0.44)	0.59 (0.49–0.71)
Change to lower major group	1.30 (1.26–1.33)	1.24 (1.2–1.28)	1.12 (1.01–1.24)	1.22 (1.08–1.37)	0.82 (0.75–0.91)	1.07 (0.95–1.20)
*Size of workplace*						
1–9 employees	1.06 (1.03–1.09)	0.96 (0.93–0.99)	1.24 (1.13–1.37)	0.95 (0.85–1.06)	1.79 (1.65–1.94)	1.42 (1.28–1.57)
10–49 employees	Ref	Ref	Ref	Ref	Ref	Ref
50–99 employees	1.02 (0.98–1.06)	1.05 (1.01–1.09)	0.73 (0.63–0.85)	0.79 (0.67–0.93)	0.66 (0.57–0.76)	0.66 (0.55–0.8)
100–499 employees	0.99 (0.96–1.02)	1.42 (1.28–1.57)	0.81 (0.72–0.92)	1.00 (0.96–1.03)	0.66 (0.58–0.75)	0.90 (0.78–1.04)
500 + employees	1.01 (0.96–1.06)	1.06 (1.00–1.12)	0.74 (0.59–0.92)	0.92 (0.72–1.18)	0.48 (0.37–0.63)	0.71 (0.52–0.95)

Men had a lower risk of belonging to any of the trajectories with SA/DP than women (OR Low SA/DP 0.42, 95% CI 0.41–0.44; Increasing SA/DP 0.34, 0.30–0.38; High SA/DP 0.33, 0.29–0.37). The risk of belonging to any of the trajectories with SA/DP was higher in the older age groups (except the oldest age group 65–67, which had a very low risk), and was highest for those aged 55–65 (OR Low SA/DP 1.34, 95% CI 1.29–1.40; Increasing SA/DP 1.36, 1.15–1.60; High SA/DP 2.40, 2.08–2.77). Those who lived outside the big cities and surrounding areas had a higher risk of belonging to trajectory with SA/DP, particularly the trajectory High SA/DP (OR Medium-sized cities 1.42, 95% CI 1.27–1.58; Small town/rural 1.85, 1.65–2.08). Those born outside the EU had a higher risk of belonging to the trajectories Increasing SA/DP (OR 1.59, 95% CI 1.31–1.94) and High SA/DP (OR 1.49; 95% CI 1.20–1.84).

Most tested associations between job demands/control and belonging to a particular trajectory were non-significant or very close to 1 after adjusting for other work-related and sociodemographic factors, except for individuals with low control having a higher risk of belonging to the trajectory High SA/DP (OR low demands/low control 1.51; 95% CI 1.25–1.83; medium demands/low control 1.47, 1.21–1.78; high demands/low control 1.35, 1.13–1.61).

People who worked at workplaces with 1–10 employees had a higher risk of belonging to the trajectory High SA/DP (OR 1.42, 95% CI 1.28–1.57) than at workplaces with 10–49 employees.

Those who had changed to a different branch of industry in 2016 than in 2012 had higher risks of belonging to the trajectory High SA/DP, especially those whose branch of industry was unknown (OR 3.5, 95% CI 2.44–5.02), care and education (OR 2.54, 95% CI 2.02–3.19), and hospitality (OR 2.49, 95% CI 1.59–3.91). Those who changed to manufacturing were not significantly different from those who remained in the trade and retain industry (OR 0.83, 95% CI 0.65–1.07).

Those who changed sector had a slightly higher risk of belonging to the trajectories Low SA/DP (municipal OR 1.32, 95% CI 1.21–1.44; region 1.26, 1.10–1.45; state 1.40, 1.32–1.48) and Increasing SA/DP (municipal OR 1.65, 95% CI 1.24–2.19, state 1.31, 1.04–1.64, other 1.64, 1.25–2.10). Those who changed to a higher occupational major group had a lower risk of belonging to the trajectory High SA/DP (OR 0.59. 95% 0.49–0.71). Other differences among those who changed occupations were small or non-significant.

The sensitivity analyses where we excluded those ≥ 61 years in 2012 showed no major differences to the main results (Suppl. Tables 1 and 2).

## Discussion

In this large population-based longitudinal cohort study of all white-collar employees in the trade and retail industry, we found four trajectories of people with similar patterns of mean annual SA and DP days during the studied seven years. The majority belonged to the trajectory No SA/DP (73%), which had zero days of SA/DP each year. The second most common trajectory was Low SA/DP (24%), characterised by those who had few SA/DP days during one or more years. The trajectories Low SA/DP and Increasing SA/DP were relatively similar to the trajectory No SA/DP on most sociodemographic and work-related factors. Job demands and control had a relatively weak association with belonging to a particular trajectory, although low control was associated with a higher risk of belonging to the trajectory High SA/DP, especially when combined with low demands. The trajectory High SA/DP had a higher proportion of women, people of higher ages, living in medium-sized or small towns, and born outside the EU. These are factors that also previously have been found to be associated with SA/DP [35, 36], although to different magnitudes in different groups. The magnitudes of the associations in this study were generally fairly small.

The level of job control has been shown to be more strongly associated with SA/DP than the level of job demands also in studies of other occupations and the general working population [37–40]. Studies within the trade and retail industry have also emphasised the importance of managing the level of job control, since that is often easier than managing the level of demands, which is driven to a large extent by customers [20].

We only found a few associations between change of occupation, branch of industry, or sector and belonging to a particular trajectory. Changing your job can be a part of work rehabilitation, especially if the work is judged to have contributed to your SA, or if there are limited options to adapt the work to the reduced work capacity [41]. A study from Sweden, based on all who were 20–60 years old and on SA since more than 180 gross days, found that those who changed their occupation were more likely to still be in paid work four years later than those who had not changed their occupation; however, among those whose SA spell had lasted 1–180 days, those who changed their occupation were less likely to be in paid work four years later than those who had not. However, it was unclear to what extent they had the possibility of work adaptations at their jobs, which may to some extent have influenced the results [41]. In our study, most of those who were white-collar workers in the trade and retail industry in 2012 were also in the trade and retail industry in 2016 (70%), and 82% were still in the private sector. However, almost half had changed major occupational group. It is thus more common to change occupation than to change branch of industry or sector. There was an association between having changed branch of industry and belonging to a trajectory with more SA/DP, especially the trajectory High SA/DP. However, we do not have information about why they changed occupation, branch of industry or sector – it is possible that this change was related to their health or morbidity, something that can be studied with information about morbidity.

This study gives more insight into different trajectories of SA/DP among privately employed white-collar workers in the trade and retail industry. The vast majority of people (97%) had either no SA/DP, or only very few days during one or a few years, which should reassure employers, physicians, insurance agencies, etc. However, more knowledge is needed on those belonging to trajectories with high or increasing SA/DP, potentially regarding causes of their SA/DP, in order to facilitate effective interventions.

### Strengths and limitations

The main strength of this study is the large and population-based cohort including all 189,321 individuals who lived in Sweden all of 2012, were 18–67 years old, and were employed in a white-collar occupation by a private company in the trade and retail industries, and who were still alive and living in Sweden in 2016. This means that the study is not based on a sample, and that the study population was large enough for subgroup analysis. Another important strength is that we could use microdata from three nationwide administrative registers of good quality [42], that there were no drop-outs (all could be followed up from inclusion to emigration, death, or end of follow-up), and that no self-reports, possibly affected by recall bias, were used. The long follow-up, stretching over seven years, is another strength, and that we can show developments and trends over time in different groups, rather than just measuring the outcome at one time point or the time to a specific event, as is traditionally done in this type of study.

Limitations are the exploratory and observational nature of the study, meaning that we are unable to draw any causal inferences from the results. That we only used SA spells > 14 days can be seen as both a strength and a limitation. Similarly, the use of a JEM to measure job demands and control can be seen as both a strength and a limitation. While the use of a JEM avoids reporting bias from people with poor work capacity potentially estimating the demands and control of their job as different from people with good work capacity, it may have introduced some misclassification of the exposure, since not all people in the same occupation have the same levels of demands and control. Many of the included factors had only a weak association with SA/DP trajectories. This indicates that there might be additional factors of importance that were not included in this study. Another limitation is that the occupational code is not updated each year for each person, meaning that we might have missed some changes of occupation.

## Conclusion

The absolute majority of the white-collar workers in the trade and retail industry had no or very low number of SA/DP days in the seven-year study period. Several sociodemographic factors were associated with belonging to a particular trajectory, however, most work-related factors were only weakly associated with most trajectories. The trajectory High SA/DP was distinct from the others on several sociodemographic and work-related characteristics, indicating that those who belong to this trajectory warrant future study and potentially interventions to prevent SA/DP.

## Supplementary Information

**Additional file 1: Supplementary Table 1.** Distribution of sociodemographic and job-related characteristics in each of the 4 trajectories of white-collar workers in the trade and retail industry 2012 with different patterns of sickness absence (SA)/disability pension (DP) days/year over 2012-2016 identified by group-based trajectory modelling, among those ≤61 years in 2012. **Supplementary Table 2.** Crude and mutually adjusted odds ratios (OR) and 95% confidence intervals (CI) for the association between sociodemographic and work-related factors and belonging to respective trajectory group of sickness absence (SA)/disability pension (DP) days per year, compared to the trajectory group called No SA/DP, among those ≤61 years in 2012.

## Data Availability

The used data cannot be made publicly available due to privacy regulations. According to the General Data Protection Regulation, the Swedish law SFS 2018:218, the Swedish Data Protection Act, the Swedish Ethical Review Act, and the Public Access to Information and Secrecy Act, these types of sensitive data can only be made available for specific purposes that meets the criteria for access to this type of sensitive and confidential data as determined by a legal review. Professor Kristina Alexanderson (Kristina.alexanderson@ki.se) can be contacted regarding the data.
