# Supplementary material for: Trajectories of sickness absence and disability pension days among 189,321 white-collar workers in the trade and retail industry; a 7-year longitudinal Swedish cohort study
Source: BMC Public Health. 2022 Aug 21;22:1592. doi: 10.1186/s12889-022-14005-y (PMC9392931; doi:10.1186/s12889-022-14005-y)
Supplement: Supplementary file 1 — Additional file 1: Supplementary Table 1. Distribution of sociodemographic and job-related characteristics in each of the 4 trajectories of white-collar workers in the trade and retail industry 2012 with different patterns of sickness absence (SA)/disability pension (DP) days/year over 2012-2016 identified by group-based trajectory modelling, among those ≤61 years in 2012. Supplementary Table 2. Crude and mutually adjusted odds ratios (OR) and 95% confidence intervals (CI) for the association between sociodemographic and work-related factors and belonging to respective trajectory group of sickness absence (SA)/disability pension (DP) days per year, compared to the trajectory group called No SA/DP, among those ≤61 years in 2012. [file 12889_2022_14005_MOESM1_ESM.docx]

Supplementary Table 1. Distribution of sociodemographic and job-related characteristics in each of the 4 trajectories of white-collar workers in the trade and retail industry 2012 with different patterns of sickness absence (SA)/disability pension (DP) days/year over 2012-2016 identified by group-based trajectory modelling, among those ≤61 years in 2012

|  | No SA/DP (n=129 656) | Low SA/DP (n=42 478) | Increasing SA/DP (n=2341) | High SA/DP (n=2648) |
| --- | --- | --- | --- | --- |
|  | **%** | **%** | **%** | **%** |
| *Total* | 73.2 | 23.98 | 1.32 | 1.5 |
|  |  |  |  |  |
| *Sex* |  |  |  |  |
| Women | 63.39 | 32.25 | 1.87 | 2.49 |
| Men | 81.09 | 17.20 | 0.84 | 0.86 |
| *Age* |  |  |  |  |
| 18-24 years | 77.82 | 21.13 | 0.74 | 0.31 |
| 25-34 years | 73.13 | 25.23 | 1.06 | 0.58 |
| 35-44 years | 74.82 | 22.65 | 1.29 | 1.24 |
| 45-54 years | 72.75 | 23.64 | 1.49 | 2.12 |
| 55-61 years | 68.72 | 26.42 | 1.58 | 3.28 |
| *Type of living area* |  |  |  |  |
| Large city | 74.16 | 23.52 | 1.18 | 1.15 |
| Medium-sized town | 73.14 | 23.77 | 1.32 | 1.78 |
| Small town or rural | 70.41 | 25.36 | 1.63 | 2.60 |
| *Education (years)* |  |  |  |  |
| Elementary (0-9 years) | 68.48 | 26.81 | 1.79 | 2.92 |
| High school (10-12 years) | 71.53 | 25.21 | 1.42 | 1.83 |
| College/university (>12 years) | 76.26 | 21.67 | 1.04 | 1.03 |
| *Birth country* |  |  |  |  |
| Sweden | 73.52 | 23.66 | 1.26 | 1.57 |
| Other Nordic country | 69.84 | 26.14 | 1.75 | 2.27 |
| Other EU25 | 73.34 | 23.43 | 1.73 | 1.49 |
| Rest of the world | 68.64 | 27.96 | 1.70 | 1.70 |
| *Family situation* |  |  |  |  |
| Married/partner without children | 70.73 | 25.18 | 1.36 | 2.73 |
| Married/partner with children | 74.79 | 22.88 | 1.16 | 1.17 |
| Single without children | 73.9 | 23.26 | 1.29 | 1.55 |
| Single with children | 62.49 | 32.47 | 2.34 | 2.70 |
| *Demands/control* |  |  |  |  |
| High demands/high control | 78.84 | 19.41 | 0.94 | 0.80 |
| High demands/medium control | 71.01 | 25.89 | 1.49 | 1.61 |
| High demands/low control | 73.72 | 23.83 | 1.22 | 1.23 |
| Medium demands/high control | 80.46 | 17.89 | 0.87 | 0.78 |
| Medium demands/medium control | 75.41 | 22.08 | 1.13 | 1.37 |
| Medium demands/low control | 64.61 | 30.77 | 1.93 | 2.70 |
| Low demands/high control | 79.90 | 18.34 | 0.89 | 0.87 |
| Low demands/medium control | 73.72 | 23.83 | 1.22 | 1.23 |
| Low demands/low control | 67.53 | 28.60 | 1.55 | 2.32 |
| *Size of workplace* | |  |  |  |
| 1-9 employees | 71.56 | 24.28 | 1.60 | 2.56 |
| 10-49 employees | 73.58 | 23.63 | 1.32 | 1.47 |
| 50-99 employees | 73.88 | 24.18 | 0.97 | 0.97 |
| 100-499 employees | 74.25 | 23.69 | 1.08 | 0.98 |
| 500+ employees | 74.25 | 24.05 | 0.98 | 0.72 |
| *Changed branch of industry in 2016* | |  |  |  |
| Construction (n=2337) | 70.73 | 26.66 | 0.98 | 1.63 |
| Hospitality (n=896) | 65.85 | 29.58 | 2.01 | 2.57 |
| Manufacturing (n=9274) | 76.28 | 22.01 | 0.79 | 0.93 |
| Unknown (n=10,676) | 65.31 | 23.03 | 4.76 | 6.89 |
| Services (n=24,103) | 73.02 | 24.70 | 1.11 | 1.17 |
| Transport (n=1217) | 69.76 | 27.61 | 0.90 | 1.73 |
| Care and education (n=6252) | 57.31 | 38.10 | 1.92 | 2.67 |
| Trade and retail (n=134,566) | 74.52 | 23.18 | 1.07 | 1.23 |
| *Changed sector in 2016* |  |  |  |  |
| Municipal (n=3880) | 58.02 | 37.50 | 2.27 | 2.22 |
| Region (n=1146) | 59.25 | 37.70 | 1.48 | 1.57 |
| State (n=5717) | 63.11 | 33.69 | 1.50 | 1.70 |
| Other (n=3477) | 69.51 | 26.17 | 1.84 | 2.47 |
| Private sector (n=158,476) | 74.96 | 22.95 | 1.00 | 1.09 |
| *Change of occupation* | |  |  |  |
| Change within occupational category or no change (n=93,999) | 73.65 | 22.93 | 1.43 | 1.98 |
| Change of occupational category within the same SSYK chapter (n=43,372) | 73.71 | 24.10 | 1.04 | 1.15 |
| Change to a higher SSYK chapter (e.g. from 2 to 1) (n=19,568) | 76.82 | 21.55 | 0.87 | 0.76 |
| Change to a lower SSYK chapter (e.g. from 1 to 2) (n=32,382) | 69.10 | 27.87 | 1.50 | 1.53 |

Supplementary Table 2. Crude and mutually adjusted odds ratios (OR) and 95% confidence intervals (CI) for the association between sociodemographic and work-related factors and belonging to respective trajectory group of sickness absence (SA)/disability pension (DP) days per year, compared to the trajectory group called No SA/DP, among those ≤61 years in 2012

|  | Low SA/DP (n=42 478) | | Increasing SA/DP (n=2341) | | High SA/DP (n=2648) | |
| --- | --- | --- | --- | --- | --- | --- |
|  | Crude OR (95% CI) | Adjusted OR (95% CI) | Crude OR (95% CI) | Adjusted OR (95% CI) | Crude OR (95% CI) | Adjusted OR (95% CI) |
| *Sex* |  |  |  |  |  |  |
| Women | Ref | Ref | Ref | Ref | Ref | Ref |
| Men | 0.40 (0.40-0.41) | 0.42 (0.41-0.43) | 0.35 (0.32-0.38) | 0.33 (0.29-0.38) | 0.26 (0.24-0.28) | 0.32 (0.28-0.36) |
| *Age* |  |  |  |  |  |  |
| 18-24 years | 0.90 (0.85-0.95) | 0.75 (0.70-0.8) | 0.54 (0.41-0.71) | 0.35 (0.25-0.50) | 0.26 (0.18-0.39) | 0.09 (0.05-0.16) |
| 25-34 years | 1.14 (1.11-1.17) | 1.11 (1.07-1.14) | 0.84 (0.74-0.95) | 0.80 (0.69-0.92) | 0.48 (0.41-0.56) | 0.40 (0.33-0.48) |
| 35-44 years | Ref | Ref | Ref | Ref | Ref | Ref |
| 45-54 years | 1.07 (1.04-1.10) | 1.1 (1.06-1.13) | 1.19 (1.08-1.32) | 1.12 (0.99-1.26) | 1.78 (1.62-1.96) | 1.89 (1.69-2.13) |
| 55-61 years | 1.33 (1.28-1.38) | 1.38 (1.32-1.44) | 1.78 (1.57-2.02) | 1.61 (1.36-1.90) | 2.87 (2.58-3.21) | 2.56 (2.20-2.98) |
| *Type of living area* |  |  |  |  |  |  |
| Large city | Ref | Ref | Ref | Ref | Ref | Ref |
| Medium-sized town | 1.02 (1-1.05) | 1.02 (0.99-1.05) | 1.14 (1.03-1.25) | 1.11 (0.99-1.24) | 1.55 (1.41-1.69) | 1.39 (1.24-1.55) |
| Small town or rural | 1.14 (1.11-1.18) | 1.11 (1.07-1.15) | 1.51 (1.35-1.68) | 1.43 (1.26-1.63) | 2.43 (2.20-2.67) | 1.85 (1.64-2.09) |
| *Education (years)* |  |  |  |  |  |  |
| Elementary (0-9 years) | 1.43 (1.37-1.50) | 1.61 (1.53-1.69) | 2.16 (1.86-2.50) | 2.08 (1.74-2.49) | 3.42 (3.00-3.90) | 2.75 (2.33-3.24) |
| High school (10-12 years) | 1.25 (1.22-1.28) | 1.33 (1.30-1.37) | 1.25 (1.22-1.28) | 1.36 (1.23-1.52) | 1.96 (1.79-2.15) | 1.68 (1.51-1.88) |
| College/university (>12 years) | Ref | Ref | Ref | Ref | Ref | Ref |
| *Birth country* |  |  |  |  |  |  |
| Sweden | Ref | Ref | Ref | Ref | Ref | Ref |
| Other Nordic country | 1.17 (1.09-1.26) | 1.05 (0.97-1.14) | 1.50 (1.17-1.92) | 1.14 (0.84-1.54) | 1.51 (1.20-1.90) | 1.10 (0.83-1.45) |
| Other EU25 | 0.98 (0.90-1.07) | 0.97 (0.89-1.06) | 1.30 (0.99-1.73) | 1.31 (0.94-1.82) | 0.94 (0.69-1.28) | 0.79 (0.53-1.18) |
| Rest of the world | 1.27 (1.21-1.34) | 1.28 (1.22-1.35) | 1.33 (1.12-1.59) | 1.51 (1.23-1.85) | 1.23 (1.03-1.46) | 1.54 (1.24-1.91) |
| *Family situation* |  |  |  |  |  |  |
| Married/partner without children | Ref | Ref | Ref | Ref | Ref | Ref |
| Married/partner with children | 0.80 (0.77-0.83) | 0.95 (0.91-1.00) | 0.62 (0.55-0.71) | 0.9 (0.76-1.06) | 0.38 (0.34-0.43) | 0.77 (0.67-0.89) |
| Single, without children | 0.82 (0.79-0.85) | 0.97 (0.92-1.01) | 0.71 (0.62-0.81) | 1.06 (0.89-1.27) | 0.48 (0.43-0.54) | 1.1 (0.94-1.28) |
| Single with children | 1.37 (1.30-1.44) | 1.26 (1.19-1.33) | 1.55 (1.31-1.83) | 1.45 (1.18-1.79) | 1.06 (0.92-1.23) | 1.13 (0.94-1.37) |
| *Demands/control* |  | 1.26 (1.19-1.33) |  |  |  |  |
| High demands/high control | 0.83 (0.79-0.87) | 0.88 (0.84-0.93) | 0.82 (0.67-1.01) | 0.88 (0.69-1.12) | 0.59 (0.47-0.73) | 0.53 (0.41-0.69) |
| High demands/medium control | 1.25 (1.19-1.31) | 0.98 (0.93-1.03) | 1.42 (1.19-1.68) | 1.17 (0.96-1.43) | 1.3 (1.1-1.54) | 1.02 (0.83-1.24) |
| High demands/low control | 1.65 (1.58-1.73) | 1.02 (0.97-1.07) | 2.03 (1.73-2.38) | 1.18 (0.97-1.44) | 2.46 (2.12-2.85) | 1.4 (1.16-1.69) |
| Medium demands/high control | 0.75 (0.72-0.79) | 0.91 (0.86-0.96) | 2.03 (1.73-2.38) | 0.99 (0.79-1.24) | 0.54 (0.44-0.66) | 0.60 (0.47-0.77) |
| Medium demands/medium control | Ref | Ref | Ref | Ref | Ref | Ref |
| Medium demands/low control | 1.42 (1.35-1.49) | 1.03 (0.98-1.08) | 1.65 (1.38-1.97) | 1.14 (0.92-1.40) | 2.13 (1.82-2.50) | 1.49 (1.22-1.81) |
| Low demands/high control | 0.77 (0.73-0.81) | 0.91 (0.86-0.96) | 0.76 (0.62-0.92) | 0.96 (0.76-1.21) | 0.56 (0.46-0.69) | 0.65 (0.51-0.84) |
| Low demands/medium control | 1.10 (1.05-1.15) | 0.99 (0.94-1.04) | 1.10 (0.92-1.32) | 1.05 (0.85-1.29) | 0.94 (0.79-1.13) | 0.87 (0.70-1.08) |
| Low demands/low control | 1.45 (1.38-1.51) | 1.02 (0.97-1.07) | 1.52 (1.28-1.80) | 1.06 (0.86-1.31) | 1.97 (1.69-2.30) | 1.54 (1.27-1.87) |
| *Size of workplace* |  |  |  |  |  |  |
| 1-9 employees | 1.07 (1.04-1.1) | 0.96 (0.94-0.99) | 1.26 (1.14-1.39) | 0.97 (0.86-1.08) | 1.82 (1.67-1.99) | 1.43 (1.29-1.59) |
| 10-49 employees | Ref | Ref | Ref | Ref | Ref | Ref |
| 50-99 employees | 1.02 (0.98-1.06) | 1.05 (1.01-1.09) | 0.72 (0.62-0.84) | 0.80 (0.67-0.94) | 0.67 (0.57-0.78) | 0.65 (0.54-0.79) |
| 100-499 employees | 1.00 (0.97-1.03) | 0.90 (0.79-1.04) | 0.81 (0.71-0.92) | 0.90 (0.79-1.04) | 0.66 (0.57-0.75) | 0.73 (0.62-0.86) |
| 500+ employees | 1.01 (0.96-1.06) | 1.07 (1.01-1.13) | 0.72 (0.58-0.91) | 0.93 (0.72-1.19) | 0.5 (0.38-0.65) | 0.71 (0.52-0.96) |
| *Changed branch of industry in 2016* |  |  |  |  |  |  |
| Construction | 1.22 (1.11-1.34) | 1.36 (1.23-1.5) | 0.93 (0.61-1.44) | 1.01 (0.63-1.61) | 1.53 (1.11-2.13) | 1.84 (1.29-2.60) |
| Hotell, restaurant | 1.46 (1.26-1.69) | 1.25 (1.06-1.46) | 2.20 (1.37-3.53) | 1.94 (1.17-3.21) | 2.55 (1.68-3.88) | 2.57 (1.64-4.04) |
| Manufacturing | 0.94 (0.89-0.99) | 1.06 (1-1.12) | 0.73 (0.57-0.93) | 0.83 (0.65-1.07) | 0.69 (0.54-0.87) | 0.80 (0.62-1.04) |
| Unknown | 1.33 (1.25-1.43) | 1.15 (0.96-1.37) | 10.71 (9.59-11.97) | 2.18 (1.34-3.55) | 11.72 (10.57-12.99) | 3.86 (2.61-5.69) |
| Sevices | 1.33 (1.25-1.43) | 1.11 (1.07-1.15) | 1.08 (0.94-1.24) | 1.13 (0.97-1.31) | 0.99 (0.87-1.13) | 1.24 (1.07-1.44) |
| Transport | 1.31 (1.15-1.49) | 1.39 (1.21-1.59) | 0.95 (0.52-1.73) | 1.15 (0.63-2.1) | 1.49 (0.94-2.36) | 1.67 (1.01-2.76) |
| Care and education | 2.18 (2.06-2.30) | 1.55 (1.44-1.68) | 2.43 (2.01-2.94) | 1.63 (1.25-2.13) | 2.86 (2.41-3.39) | 2.52 (2.00-3.19) |
| Trade and retail | Ref | Ref | Ref | Ref | Ref | Ref |
| *Changed sector in 2016* |  |  |  |  |  |  |
| Municipal | 2.13 (1.99-2.28) | 1.31 (1.2-1.43) | 2.97 (2.39-3.71) | 1.62 (1.22-2.16) | 2.63 (2.10-3.30) | 0.96 (0.72-1.27) |
| Region | 2.07 (1.83-2.34) | 1.24 (1.08-1.43) | 1.89 (1.17-3.07) | 1.12 (0.66-1.90) | 1.88 (1.18-3.01) | 0.81 (0.48-1.35) |
| State | 1.74 (1.64-1.84) | 1.39 (1.31-1.48) | 1.82 (1.46-2.27) | 1.32 (1.05-1.66) | 1.91 (1.55-2.36) | 1.3 (1.04-1.62) |
| Other | 1.25 (1.16-1.35) | 1.07 (0.99-1.16) | 2.01 (1.55-2.61) | 1.59 (1.22-2.07) | 2.61 (2.09-3.26) | 1.66 (1.32-2.1) |
| Private enterprise | Ref | Ref | Ref | Ref | Ref | Ref |
| *Change of occupation* |  |  |  |  |  |  |
| No change or change within sub-major group group | Ref | Ref | Ref | Ref | Ref | Ref |
| Change of sub-major group within major group | 1.04 (1.02-1.07) | 1.00 (0.97-1.03) | 0.68 (0.61-0.76) | 0.82 (0.72-0.93) | 0.58 (0.52-0.64) | 0.78 (0.69-0.87) |
| Change to higher major group | 0.90 (0.86-0.93) | 0.87 (0.83-0.9) | 0.55 (0.47-0.65) | 0.68 (0.57-0.82) | 0.36 (0.30-0.43) | 0.56 (0.46-0.67) |
| Change to lower major group | 1.29 (1.25-1.33) | 1.24 (1.2-1.28) | 1.07 (0.96-1.19) | 1.22 (1.08-1.39) | 0.83 (0.75-0.92) | 1.05 (0.93-1.19) |
